# Supplementary material for: Adherence to Covid-19 preventive measures among high school students in Jimma town, South-West Ethiopia: Institutional-based cross-sectional study
Source: PLoS One. 2022 Dec 30;17(12):e0279081. doi: 10.1371/journal.pone.0279081 (PMC9803269; doi:10.1371/journal.pone.0279081)
Supplement: S2 File — (DOCX) [file pone.0279081.s003.docx]

| S.N | Questions | | Possible answers |
| --- | --- | --- | --- |
| **I. students socio-demographic characteristics** | | | |
|  | Age of the students | | _____years |
|  | Sex of the student | | 1. Female  2. Male |
|  | Ethnicity | | 1. Oromo 2. Amahara 3. Tigre 4. Gurage 5. Others (specify) |
|  | Religion | | 1. Orthodox 2. Muslim 3. Protestant 4. Othres (Specify) |
|  | Educational Status | | 1. Grade 9 2. Grade 10 |
|  | students residency | | 1.Urban  2.Rural |
|  | History of medical illness | | 1. Yes (if yes go to #8).  2. no |
|  | Types of medical illness | | 1. DM 2. HTN 3. Asthma 4. pulmonary TB 5. Other (specify) |
| II. | **Institution-related characteristics of students in selected public schools** | | |
|  | Presence of functional hand washing facility at the main entrance gate of school | 1. Yes 2. No | |
|  | Presence of water and soap at all the time | 1. Yes 2. No | |
|  | Presence of written material promoting hand washing posted next to the hand washing facility | 1. Yes 2. No | |
|  | Presence of posters or written materials displayed at various corners school promoting hand washing, respiratory hygiene, and physical distancing | 1. Yes 2. No | |
| 13. | Arrangement of tables and chairs in a manner that the distance from the back of one chair to the back of another chair be at least 2 meters apart | 1. Yes 2. No | |
| 14. | Adequate ventilation (ie opening windows and installing AC ventilators) is ensured in areas of potential gathering such as corridors, staff works rooms, classrooms. | 1. Yes 2. No | |
| 15. | Teachers are provided with face masks to be worn during their regular working hours and shift changes | 1. Yes 2. No | |
| 16. | Management of the school provided education or training for all workers on COVID-19 and how to prevent it. | 1. Yes 2. No | |
| III | **Wash hands after coughing/sneezing** |  | |
| 17. | Frequency of hand sanitation (using soap, liquid soap, or alcohol-based sanitizer) | - 1. Every time   2. Often   3. Sometime   4. Never | |
| 18. | Avoid touching face | 1. yes 2. No | |
| IV. | **Face mask /Respiratory hygiene** |  | |
| 19. | Frequency of face mask wearing in the school | - 1. Every time   2. Often   3. Sometimes   4. Never | |
| 20. | Wear a face mask when leaving home | 1. yes 2. No | |
| 21. | Consistent face mask wearing in any public space/class | 1. yes 2. No | |
| 22. | Type of facemask worn | 1. Respirators 2. Surgical masks 3. Cloth face coverings 4. Respirators and Surgical masks 5. Surgical masks and Cloth face coverings | |
| 23. | Re-use of facemasks | 1. Yes 2. No | |
| V. | **Social distancing** /**Physical distancing/isolation** |  | |
| 24. | Avoiding social and meal gatherings with other people who do not live together | 1. Yes 2. No | |
| 25. | Avoiding crowded place | 1. Yes 2. No | |
| 26. | Follow rule of staying 1.5-2m from other people | 1. Yes 2. No | |
| 27. | Measure temperature twice a week | 1. Yes 2. No | |
| 28. | Stay home when experiencing flu-like symptoms (among people who had flu-like symptom days | 1. Yes 2. No | |
| **VI Respondents’ knowledge of the novel coronavirus infection** | | | |
| 29. | 1. Can COVID-19 present with symptoms such as fever, tiredness, dry cough and dyspnea | | 1.Yes  2.No |
| 30. | Unlike the common cold, stuffy nose, runny nose, and sneezing are less common in persons infected with the COVID-19 virus | | 1.Yes  2.No |
| 31. | There currently is no effective cure for COVID-2019, but early symptomatic and supportive treatment can help most patients recover from the infection | | 1.Yes  2.No |
| 32. | Not all persons with COVID-2019 will develop to severe cases. Only those who are elderly, have chronic illnesses, and are obese are more likely to be severe cases. | | 1.Yes  2.No |
| 33. | Eating or contacting wild animals would result in the infection by the COVID-19 virus | | 1.Yes  2.No |
| 34. | Persons with COVID-2019 cannot infect the virus to others when a fever is not present | | 1.Yes  2.No |
| 35. | The COVID-19 virus spreads via respiratory droplets of infected individuals | | 1.Yes  2.No |
| 36. | Ordinary residents can wear general medical masks to prevent the infection by the COVID-19 virus | | 1.Yes  2.No |
| 37. | It is not necessary for children and young adults to take measures to prevent the infection by the COVID-19 virus | | 1.Yes  2.No |
| 38. | To prevent the infection by COVID-19, individuals should avoid going to crowded places such as train stations and avoid taking public transportations | | 1.Yes  2.No |
| 39. | Isolation and treatment of people who are infected with the COVID-19 virus are effective ways to reduce the spread of the virus | | 1.Yes  2.No |
| 40. | People who have contact with someone infected with the COVID-19 virus should be immediately isolated in a proper place | | 1.Yes  2.No |
|  | One way of prevention of COVID 19 is not touching the eye, nose with unwashed hands. | | 1.Yes  2.No |
|  | Frequent and proper handwashing with soap and water is one method of COVID-19 prevention. | | 1.Yes  2.No |
| VII | **Attitudes of the respondents towards COVID-19 preventive measures** | |  |
| 41. | 1. COVID-19 is a killer disease. COVID-19 is preventable. | | 1.Strongly disagree  2. Disagree  3. Neutral  4. Agree  5.Strongly agree |
| 42. | 1. COVID-19 is preventable | | 1.Strongly disagree  2. Disagree  3. Neutral  4. Agree  5.Strongly agree |
|  | 1. Government is responsible for implementing the preventive measures of COVID-19. | | 1.Strongly disagree  2. Disagree  3. Neutral  4. Agree  5.Strongly agree |
|  | 1. Community is responsible for implementing preventive measures of COVID-19. | | 1.Strongly disagree  2. Disagree  3. Neutral  4. Agree  5.Strongly agree |
|  | Individuals are responsible to apply all the preventive measures of COVID-19 | | 1.Strongly disagree  2. Disagree  3. Neutral  4. Agree  5.Strongly agree |
| VIII. | **Covid-19 exposure status related factors** | | 1. Yes 2. No |
| 43. | Have you ever been a case of COVID-19? | | 1. Yes 2. No |
| 44. | Have any member of your family been a case of COVID-19? | | 1. Yes 2. No |
| 45. | Have any of your friends been a case of COVID-19? | | 1. Yes 2. No |
| 46. | Did any member of your family die because of COVID-19? | | 1. Yes 2. No |
| 47 | Did any member of your friends die because of COVID-19? | | 1. Yes 2. No |
