# Supplementary material for: Adherence to Covid-19 preventive measures among high school students in Jimma town, South-West Ethiopia: Institutional-based cross-sectional study
Source: PLoS One. 2022 Dec 30;17(12):e0279081. doi: 10.1371/journal.pone.0279081 (PMC9803269; doi:10.1371/journal.pone.0279081)
Supplement: Attachment — Submitted filename: Comment plos one.docx [file pone.0279081.s004.docx]

Comment #1: Title

Title: ADHERENCE TOWARDS COVID-19 PREVENTIVE MEASURES AND ASSOCIATED FACTORS AMONG JIMMA TOWN HIGH SCHOOL STUDENTS, SOUTH-WEST, ETHIOPIA/2021: INSTITUTIONAL-BASED CROSS-SECTIONAL STUDY

Better to modify as: ADHERENCE TO COVID-19 PREVENTIVE MEASURES AMONG HIGH SCHOOL STUDENTS IN JIMMA TOWN, SOUTH-WEST ETHIOPIA: INSTITUTIONAL-BASED CROSS-SECTIONAL STUDY

Comment #2: Abstract

Background: The adherence to COVI-19….correct as COVID-19

What is known about the magnitude of COVID-19 in Africa or Ethiopia???

What is known about the adherence to COVID-19 preventive measures in Africa or Ethiopia???

Objective: covid-19…replace with COVID-19 through the manuscript, Ethiopia/2021…use comma

Methods: Institutional based cross-sectional study was conducted on systematically selected high school students attending Jimma town public high schools from July/2021-August/2021…full of repetition of some words or phrases; need revision??

Under methods mention your sample size, and data collection method.

Finally; Significant factors were performed at a significance level of <0.05…use declared than performed.

Result: … About 205(52.8%)… add confidence interval.

Conclusion: In this study the level of adherence to covid-19 preventive measures was low…what is your base to say low?? How 52.8% belongs to be low??

Your recommendation is not in line with your findings???

**Comment #3: Introduction**

The outbreak was first identified in December 2019 in Wuhan, China…this is not new for readers??

Largest disruption of education systems in history…repeatedly written in 2^nd^ and 3^rd^ paragraph.

Fifth paragraph is too long try to minimize it.

**Comment #4: Methods and Materials**

**Currently, is there** preparatory school in Ethiopia??

The duration of the study was From July 2021 to August 2021…mention date??

They were illegible for the study…is it illegible or eligible??

Operational definitions: try to consider three categories (poor, moderate and good) for knowledge, and adherence???

How many items or questions were used to assess knowledge, attitude and adherence???

Why ordinary logistic regression modeling was used???

Comment #5: Results

What is the difference b/n practice and adherence??

In identifying associated to adherence such as factors knowledge and attitude, why you used poor knowledge and unfavorable attitude comparison category than good knowledge and favorable attitude??
