# Supplementary material for: Adherence to Covid-19 preventive measures among high school students in Jimma town, South-West Ethiopia: Institutional-based cross-sectional study
Source: PLoS One. 2022 Dec 30;17(12):e0279081. doi: 10.1371/journal.pone.0279081 (PMC9803269; doi:10.1371/journal.pone.0279081)
Supplement: Attachment — Submitted filename: Response to Reviewers.docx [file pone.0279081.s005.docx]

**POINT-BY-POINT RESPONSE TO THE COMMENTS FROM REVIEWERS**

**Dear:Jianhong Zhou (Staff EditorPLOS ONE)**

First, we would like to appreciate your timely response to our submission. Generally, we found the questions and comments from the reviewers are interesting and appropriate. We accept the comments and suggestions. We have gone through based on reviewer comments and suggestions and we have made corrections accordingly.

We are pleased to submit the improved manuscript .Changes and modifications are highlighted in the revised version of the manuscript. A point-by-point response to the comments and questions was given as stated hereunder.We used the same letter to write our responses.

**Modified Title:** ADHERENCE TO COVID-19 PREVENTIVE MEASURES AMONG HIGH SCHOOL STUDENTS INJIMMA TOWN, SOUTH-WEST ETHIOPIA: INSTITUTIONAL-BASED CROSS-SECTIONAL STUDY

| **Sections** | **Reviewers Questions and Suggestions** | | **Authors actions and Response** |
| --- | --- | --- | --- |
| **REVIEWER #1** | | |  |
| **Comment #1: Title** | | **Better to modify as:**  ADHERENCE TO COVID-19 PREVENTIVE MEASURES AMONG HIGH SCHOOL STUDENTS INJIMMA TOWN, SOUTH-WEST ETHIOPIA: INSTITUTIONAL-BASED CROSS-SECTIONAL STUDY | **Dear reviewer thank you for your suggestion**  We accept your suggestions and we have made an appropriate correction/modification in the revised manuscript. |
| **Comment #2: Abstract** | | **Background:** |  |
|  |  | 1. The adherence to COVI-19….correct as COVID-19 | **Great! We appreciate your comment.**  We accept your suggestions and it has been corrected accordingly. |
|  |  | 2. What is known about the magnitude of COVID-19 in Africa or Ethiopia??? | **Thank you for your concern**  We accept your concern and we had added additional information about the magnitude in Africa and in Ethiopia as follows:  According the world meter live report, confirmed cases were 8,654,048 cases, 221,941 deaths and 8,035,451 cases were recovered from COVID-19 in Africa and 369,867 cases, 6,662 deaths and 346,547 recovery were recorded in Ethiopia. |
|  |  | 3. What is known about the adherence to COVID-19 preventive measures in Africa or Ethiopia??? | **Thank you for your insightful questions**  We share your concerns and we had added additional information in the revised manuscript which stated as:  World Health Organization (WHO) has recommended several preventive measures of COVID-19 and African countries including Ethiopia had accepted and engaged in recommended preventive measures. As part of these measures, the use of face masks, hand washing, physical distancing, cough etiquette and avoidance of crowded places are recommended |
|  |  | **Objective:** |  |
|  |  | 1. covid-19…replace with COVID-19 through the manuscript, Ethiopia/2021…use comma | **We would like to thank you for important suggestion**  We share your suggestion and we have made corrections throughout the document in the revised manuscript. |
|  |  | **Methods:** |  |
|  |  | 4. Institutional based cross-sectional study was conducted on systematically selected high school students attending Jimma town public high schools from July/2021-August/2021…full of repetition of some words or phrases; need revision?? | **Thank you for your insightful comment**  We agree with your concerns, we have made a revision, and repetition of some words has been removed.  in the revised manuscript currently it has been written as:  **Methodsand Materials:**  An institution-based cross-sectional study was conductedamong 404 systematically selected high school students of Jimma townfrom July 15toAugust 2, 2021.  The sample size was determined by using a single population proportion formulaand Data were collected through face-to-face interviews using a structured and pretestedquestionnaire. |
|  |  | 5. Under methods, mention your sample size, and data collection method. | **Thank you for your important suggestion**  We accept your suggestion and we have added what you suggested. |
|  |  | 1. Finally; Significant factors were performed at a significance level of <0.05…use declared than performed | **Thank you!**  We accept your suggestions and it has been corrected in the revised manuscript |
|  |  | **Result:** |  |
|  |  | 1. …About 205(52.8%)… add confidence interval | **We would like to thank you for your critical issue.**  we accept your indication and we had made corrections on revised manuscript and currently it states as:  From 388 students included in the analysis about 52.8% (95%CI:0.4783-0.5779) of students had good level of adherence towards covid-19 preventive measures |
|  |  | **Conclusion:** |  |
|  |  | 1. In this study the level of adherence to covid-19 preventive measures was low…what is your base to say low?? 2. How 52.8% belongs to be low?? | **Dear reviewer we really appreciate your question,**  We really feel regret to do mistakes on reporting in such alike ways, this was actually done with lack of understanding how to report.  Now we agree to correct our mistakes and we would like to correct it. Our bases to say evidences did not support low, but now we had made corrections accordingly using results of previously conducted papers.  Our study finding (52.8%) is higher than the studies conducted among Gondar City residents, Dirashe District, Southern Ethiopia and other studies.  therefore we have made corrections on our conclusions in revised manuscript as :  **Conclusion**: In this study, adherence to covid-19 preventive measures was high in the areathan the other local and national figures.Being male, lack of access to water and soap, unfavourable attitude and poor knowledge were associated factors with adherence to covid-19 preventive measures. Therefore, to ensure maximal adherence to preventive measures for COVID-19, special strategies should focus on their adoption by male students, access to water and soap, the concerned bodies should implement, activities to increase knowledge and change attitude on COVID-19 preventive measures. |
|  |  | 2. Your recommendation is not in line with your findings??? | **Thank you.**  We have made revisions on our recommendation section of our conclusion in line with our findings. |
| **Comment #3: Introduction** | | 1. The outbreak was first identified in December 2019 in Wuhan, China…this is not new for readers?? | **Thank you**  we have made corrections and rearrangements in the revised manuscript |
|  |  | 2. Largest disruption of education systems in history…repeatedly written in 2^nd^ and 3^rd^ paragraph. | **We appreciate you!**  Corrected accordingly in the revised manuscript |
|  |  | 3. Fifth paragraph is too long try to minimize it | **Thank you**  It has been corrected in the revised manuscript and highlighted |
| **Comment #4: Methods and Materials** | | 1. **Currently, is there** preparatory school in Ethiopia?? | **Thank you!**  Dear reviewer thank you for your important indication to be corrected, currently there is no preparatory school in Ethiopia. They are changed to higher secondary schools. We had made corrections in the revised manuscript |
|  |  | 2. The duration of the study was From July 2021 to August 2021…mention date?? | **Thank you for you suggestion**  We had made corrections in the revised manuscript as:  The study was conducted from July 15 to August 2, 2021. |
|  |  | 3. They were illegible for the study…is it illegible or eligible?? | **Thank you your insightful review**  It has been corrects in the revised manuscript as **:eligible** |
|  |  | **Operational definitions**: |  |
|  |  | 1. Try to consider three categories (poor, moderate and good) for knowledge, and adherence??? | **Thank you for your important suggestion**  We appreciate your suggestion and commitment; unfortunately we had collected our data using questionnaires configured as**yes/no,** for both knowledge and adherence, but not likeret it was actually done according to references done before our study.  Your suggestion is important if we had collected the data using questionnairesconfigured in the form of Likert  scale, but currently it is impossible,because Generaladherence to the COVID-19 preventive measures was assessed using items configured as **yes/no** questions in the questionnaire, generated from hand washing, using a facemask, keeping physical distance, not travel to a crowded place, home stay, and not travel to anyplace out of the city in the last 14 days.  Hence, an individual was considered as **having good** adherence towards COVID-19 preventive measures if he/she was able to answer **‘yes’** to the median and above of the aforementioned composite variables.  **Therefore,** we authors agree not to change the category of variables because of the data were not collected according to your suggestion. But we appreciate your suggestion that will be considered for our future research activity.  **Thank you again for understanding us.** |
|  |  | 2. How many items or questions wereused to assess knowledge, attitude and adherence??? | **Thank you for your insightful questions**  We accept and we had added the number of questionnaires in the **operational definition** of revised manuscript including they how we had scored them**. (See the operational definition)**  **Adherence**=6 questions  **Attitude** =5 questions  **Knowledge** =14 questions |
|  |  | 3. Why ordinary logistic regression modelling was used??? | **Thank you for asking very important question**  **Dear reviewer,** thiswas written mistakenly due to lack of understanding of the errors done during typing (typing error).we would like to appreciate you for asking scientific question which is critical, but we would like to ask an apology of this mistake with regret, what we actually done was differ from what we wrote. As it was mention in abstract section of our manuscript we had made Bivariate and **multivariabl**e logistic regression analysis to identify significant variables, since our outcome variable was aggregated as good and poor level of adherence(binary outcome), which is not ordinal , therefore we didn’t conduct ordinary logistic regression modelling.  Because, this ordinary logistic regression used to model the relationship between an ordinal response variable and one or more explanatory variables. An ordinal variable is a categorical variable for which there is a clear ordering of the category levels. |
| **Comment #5: Results** | | 1. What is the difference b/n practice and adherence?? | **Thank you for your question**  The difference between practice and adherence is expressed as follows:  Both terms seem similar but they are different.  Practice is **The act of doing something again and again in** actual performance, which may or may not according to the guide line /recommendation  **While**  **Adherence** has been defined as “**the extent to which a** person's /participants behaviour/ act, actions corresponds with agreed recommendations, government directions to prevent COVID-19or recommendations from WHO and a health care provider”.  Adherence is a more positive, proactive behaviour, which results in a lifestyle change by the peoples. |
|  |  | 2. In identifying associated to adherence such as factors knowledge and attitude, why you used poor knowledge and unfavourable attitude comparison category than good knowledge and favorable attitude?? | **We would like to thank you for your important question**  We have used poor knowledge and unfavourable attitude comparison categorythan good knowledge and favorable attitude, because we had used **Base outcome (good**), if we had used poor as an **outcome (poor)** the reverse was possible.  Now we agree to put as it is, no change was made in the revised manuscript and we try to indicate the base of outcome in the revised manuscript. |
| **Summary:** we authors feel that the reviewer, questions, comments and suggestions are very important and have a great contribution to the improvement of the paper. We have made corrections and we try to fix the typographical and language edition. This corrections and changes are highlighted with green. **Thank you again!** | | | |
