# Supplementary material for: Adherence to Covid-19 preventive measures among high school students in Jimma town, South-West Ethiopia: Institutional-based cross-sectional study
Source: PLoS One. 2022 Dec 30;17(12):e0279081. doi: 10.1371/journal.pone.0279081 (PMC9803269; doi:10.1371/journal.pone.0279081)
Supplement: Attachment — Submitted filename: Response to reviewers.docx [file pone.0279081.s006.docx]

**POINT-BY-POINT RESPONSE TO COMMENTS FROM THE REVIEWERS**

**Dear:** Md. Tanvir Hossain (Academic Editor of the PLOS ONE)

First, we thank you for your response to our submission. In general, we found that the questions and comments from the reviewers were interesting and appropriate. We accept the comments and suggestions. Based on the reviewers comments/ suggestions and we have made corrections accordingly.

We are pleased to submit an improved version of the manuscript .The changes and modifications have been highlighted in the revised version of the manuscript. A point-by-point response to the comments and questions is provided below. We have used the same letter to write our responses.

**ID:** PONE-D-21-31222R1

**Title:** ADHERENCE TO COVID-19 PREVENTIVE MEASURES AMONG HIGH SCHOOL STUDENTS IN JIMMA TOWN, SOUTH-WEST ETHIOPIA: INSTITUTIONAL-BASED CROSS-SECTIONAL STUDY

| **Sections** | **Reviewers Questions and Suggestions** | | **Authors actions and Response** |
| --- | --- | --- | --- |
| **REVIEWER #1;** Comment #2 | | |  |
| **Abstract** | | **Background:** |  |
|  |  | Indicate what is the gap with adherence to COVID-19 preventive measures among high school students in Africa or Ethiopia??? | Great! We appreciate your comment.  In the abstract of our revised manuscript we had added the statement under here:  This study conducted to fill the information gap on level of adherence to COVID-19 preventive measures among students. |
|  |  | Please remove number of case of COVID-19 in Africa /Ethiopia from background and you can explain it under introduction. | **Thank you very much**  We agree with your important suggestion, we had removed. |
|  |  | line number 37: punctuation error | **Great! we appreciate you.**  **It is now corrected/fixed in the revised manuscript** |
|  |  | **Result:** |  |
|  |  | 1. 95% CI: 0.4783- 39 0.5779…write confidence intervals as percent?? AOR: 0.57 (95% CI: 0.37-0.89) write all as (AOR = 0.57, 95% CI: 0.37-0.89)??? | **We would like to thank you for your critical issue.**  We accept your indication and we had made corrections on revised manuscript. |
|  |  | 2. Majority of the studies uses good knowledge an positive attitude, why you consider the reverse one?? | **We appreciate your concern!**  Now we had made corrections according to the majority of the studies reporting style and based on your suggestion in our revised manuscript |
|  |  | **Conclusion:** |  |
|  |  | From where you get national figure, is there systematic review??? How much is it? | **Great! Thank you for your question!**  It means from the researchers conducted in other parts of Ethiopia, but currently systematic review and meta-analysis result Published: October 13, 2022. Which shows The pooled estimate of adherence to COVID-19 preventive measures in Ethiopia was 41.15%, which is higher than our study finding. |
|  |  | What are special strategies???  Is the access to water and soap, only for male students??? | **Thank you**  It is to mean implementing strategies such as increasing, access to health information mainly targeting male students, access to water and soap to school level.  Water and soap access is **not** only for male students, it is for all school students. |
| **Comment #3** | | **Introduction** |  |
|  |  | 1. Try to minimize one page and half. | **Thank you for your appropriate suggestion!**  We had made corrections |
| **Comment #4** | | Comment #4: Methods and Materials |  |
|  | | This was my critical comment, but the author fails to revise it?? It is illogical to say 90% and 60% equally as good adherence, for 60% better say moderate?? | **Thank you!**  Dear reviewer! Thank you for your important indication how it could be corrected. We authors agree and we had made all corrections based on your indication in the revised manuscript. |
|  |  | 2. The appropriate model for these types of category is ordinary logistic regression?? | **Thank you for your suggestion**  We had made analysis again using ordinary logistic regression after your second comment, now all associations made using ordinary logistic regression model again. |
|  |  | **Operational definitions**: |  |
|  |  | 1. Try to consider three categories (poor, moderate and good) for knowledge, and adherence??? | **Thank you for your important suggestion**  **Therefore,** we authors agree to change the category of variables according to your suggestion, and we had made the regression based on the three category. The modifications had experienced on the results.  **Thank you again for understanding us.** |
| **REVIEWER #2;** Comment #1 | | | **Authors actions and Response for each specific issues** |
| This is a timely and relevant study by authors about COVID-19 among students.  The findings could be useful at the local and national levels to combat COVID-19.  My review is based on the scientific merit of the manuscript and not based on the potential implications of the paper | | | **Thank you very much** |
| **Comment** | **Methods:** | |  |
|  | Authors have mentioned that they used previously used tools to collect the knowledge, attitude, and practice data about COVID-19. The authors did not mention the validity and reliability of the tools/questionnaires. | | **Thank you for raising important point that was missed,**  **we had made additional explanations, as follows**  **Data collection instrument and quality control**  After reviewing different studies done before (1,2,14–16) the questionnaire was adapted to address the objectives of the study. To assure the quality of the data, the tool was prepared first in English and then translated into the local language (Amharic and Afan Oromo) by language experts in English and both local languages. **The reliability and validity of the instrument was checked using Cronbach alpha (0.751) and subject expertise (public health $ nursing)** respectively. A pre-test was done on 5% of the total sample size in another school, which was not selected for actual data collection. Then modifications such as wording, rephrasing, adding and deleting of some information for clarity were made on the tool accordingly. Data collectors and supervisors were trained on the data collection process for one day. The data were checked for completeness and consistency of information by the principal investigator. |
|  | **Result:** | |  |
|  | The authors have found some rather interesting/plausible results.  The univariate and adjusted logistic regression models revealed results in different directions. | | We appreciate your insight full comments!  Dear reviewer, apologize! We had made check-up to find how we made mistakes during analysis, and now we corrected and fix it in the revised manuscript.  Thank you again |
|  | There is a major flaw in the interpretation of the results.  For example in Table 6 page 15-16; Education status; Grade 9 students had higher odds of adherence to preventive measures compared to grade 10 students.  The authors have interpreted it in the other direction. | | **Thank you dear reviewer for your critical/insightful observation**  We authors note your point and we agree you are right.  we had made all the analysis again using three categories to the outcome variable according to suggestions from other(1^st^) reviewer, we feel that now your issue has been solved in the revised manuscript,  **Thank you again**! |
|  | Also, since the 95%CI excludes 1, the p-value must be <0.05.  Similarly, the authors have misinterpreted the adjusted odds ratio (most of the no-category have higher odds ratios compared to yes categories. Am I missing anything? | | **Great, we appreciate all concerns and we share it.**  We had made all the analysis again and now it is corrected based on the recommendations from both reviewers in the revised manuscript**. Table 7.** |
|  | Please pay attention to your results, when you got an odds ratio of 0.57, you said men had lower odds of adherence; which is correct, but when you got an odds ratio of 1.6 for grade 9, shouldn’t you interpret it as l- grade 9 students have 1.6 times higher odds of adherence compared to grade 10?  If you do so, you cannot say that higher education is associated with higher odds of adherence.  Also, what educational contents regarding COVID/Infectious disease might differ among grade 9 and grade 10 students? Expanding it might help discussion and conclusion. | | **We thank you!**  We try to fix all the issues that you indicate in the revised manuscript after running the statistical analysis again.  We re-write all the results and discussions based on the new statistical analysis result.  From the new statistical analysis report educational status is not associated with level of adherence, so all things are solved. |
|  | Also, please interpret your measure of association as an odds ratio. For example, you can mention.  The odds were 47%less etc..You have interpreted your odds ratios and risk ratio by directly saying that “…… 2.11 times more likely………..”. You should mention that the ratio is in odds and not directly measured as incidence. | | It is now fixed based on your recommendation in the revised manuscript  **Thank you again!** |
|  | Table 2, Page 13, the institutional factors varied according to the schools rather than the individual participants. You have used variables also in the multivariable analysis (please mention if you assume that students within the same school can have varying values of these characters, if so please justify?) There are other measures to analyze the ecological nature of data | | **Thank you for raising important point!**  We authors did not assume that students within the same school could have varying values of these characters; unfortunately, the study was conducted at different schools, so students from different schools may have different institutional factors. In addition, we have used variables in the multivariable analysis after reviewing of many other studies done previously.  Here we are ready to correct if we did mistakes again and ready to hear from you.  **Thank you again!** |
| **Minor comments** | | |  |
|  | The adherence level according to covariates can be best displayed with percentages rather than actual numbers. | | **Thank you**  accepted and corrected in the revised manuscript |
|  | Figure 1: It can be explained in a sentence and I don’t think having a figure makes it easier to understand for readers. | | **Great**  It has been corrected and submitted separately |
|  | Line 258, page 15: The word “multivariate analysis” is not suitable to explain the data analysis in this study. Please use the term adjusted/multiple/or multivariable logistic regression analysis. | | **Thank you very much**  now it is corrected |
|  | **Discussion:** | |  |
|  | I would suggest your reply to my comments about the result section. I think you need to change your discussion significantly after reviewing your results. | | **Thank you again for suggestion**  we have made changes according to the results changed accordingly |
|  | The limitation section is poorly written. It is very clear that this is a cross-sectional study and the results could not infer causality. There could be other limitations like the limitation/weakness of the questionnaire if there is any bias due to social desirability. Do you think there is a possibility of recall bias since you asked questions about having COVID to themselves, their family, and their friends?  Also, they developed a cut-off value for knowledge, attitude, and preventive practices based on half score in each category. This can create some limitations to their measure | | **we would like to appreciate all your points raised**  We had made some modifications according to your suggestion.  now the limitation section rewritten as :  The following limitation can be considered in relation to this study. It is very clear that this is a cross-sectional study and the results could not infer causality. Social-desirability bias may one limitation that students might respond to the socially acceptable responses despite their actual practice is poor. There is also a possibility of recall bias since we asked questions about having COVID-19 to themselves, their family, and their friends. In addition, it has been developed a cut-off value for attitude of respondents based on half score in each category, this can create some limitations to their measure. |
|  | **Conclusion** | |  |
|  | The conclusion is generic and not fully supported by the author's findings. They could be more specific on what implication this study holds to contribute to the battle against COVID-19 in Ethiopia and in their local settings. They found grade 9 students have higher odds of adherence compared to grade 10, why this happened. Given the scenario, how more educational programs could be helpful? | | **Thank you for your important points.**  We re-written the entire conclusion again and modified in the revised manuscript according to your suggestion. |
| **Summary:** we authors feel that the reviewer, questions, comments and suggestions were very important and have a great contribution to the improvement of the paper. We have made corrections and we try to fix the typographical and language edition. This corrections and changes highlighted with green colour .  **Thank you again!** | | | |
